# The complete chloroplast genome sequence of *Astragalus melilotoides* (Fabaceae), a leguminous forage in Northern China

**DOI:** 10.1080/23802359.2022.2164702

**Published:** 2023-01-13

**Authors:** Zhanjun Wang, Ying Tian, Bo Ji, Wangsuo Liu

**Affiliations:** a Institute of Forestry and Grassland Ecology, Ningxia Academy of Agriculture and Forestry Science, Yinchuan, China; b Ningxia Key Laboratory of Desertification Control and Soil and Water Conservation, Ningxia Academy of Agriculture and Forestry Science, Yinchuan, China; c School of Resources Environment and Life Sciences, Ningxia Normal University, Guyuan, China; d Ningxia Technical College of Wine and Desertification Prevention, Yinchuan, China

**Keywords:** *Astragalus melilotoides*, chloroplast genome, phylogenetic analysis

## Abstract

*Astragalus melilotoides* Pall. 1776 is a perennial leguminous forage, widely distributed in northern China, with cold, drought and disease resistance characteristics. Here, we determined the complete chloroplast (cp) genome sequence of *A. melilotoides.* It was 123,827 bp in length and 36.97% GC content with IR loss. The cp genome contained 110 complete genes, including 76 protein-coding genes, 30 tRNA genes, and four rRNA genes. A maximum likelihood (ML) phylogenetic tree revealed that *A. melilotoides* was related to *A. americanus*, *A. gummifer*, *A. mongholicus*, *A. nakaianus*, *A. mongholicus* var. *nakaianus*, and *A. membranaceus* var. *membranaceus*. The cp genome analysis of *A. melilotoides* will provide a reference for the phylogenetic study of *Astragalus* in the future.

## Introduction

*Astragalus melilotoides* Pall. firstly discovered by Pall. in 1776 (https://www.tropicos.org/name/Search?name=Astragalus melilotoides), is a perennial leguminous forage widely distributed in desert steppe regions in northern China (Fu 1993). Due to its characteristics of drought-, cold-tolerance, water-holding capacity, disease resistance, and feeding value, *A. melilotoides* has been used in grassland improvement and forage supplementation (Fu 1993; Hou and Jia 2004). In order to improve the palatability, seed quality and yield of *A. melilotoides*, in the last decades, some scholars established a protoplast regeneration system to ensure its feeding quality (Hou and Jia 2004), and to explore the contribution of *Agrobacterium rhizogenes* to protoplasm regeneration (Zhang et al. 2008). The excellent characters of wild *A. melilotoides* can be cultivated by means of gene editing or somatic hybridization, these are crucially mediated by *Agrobacterium rhizogenes* (Zhang et al. 2008). Above studies laid a solid foundation for the feeding prospect of *A. melilotoides.* The value of *A. melilotoides* will be paid more and more attention in future, however, studies on the phylogeny of *A. melilotoides* remains limited. We reported the complete chloroplast (cp) genome sequence of *A. melilotoides* to provide reference for further study of the phylogenetic status in Fabaceae family.

## Materials

**Figure 1.**
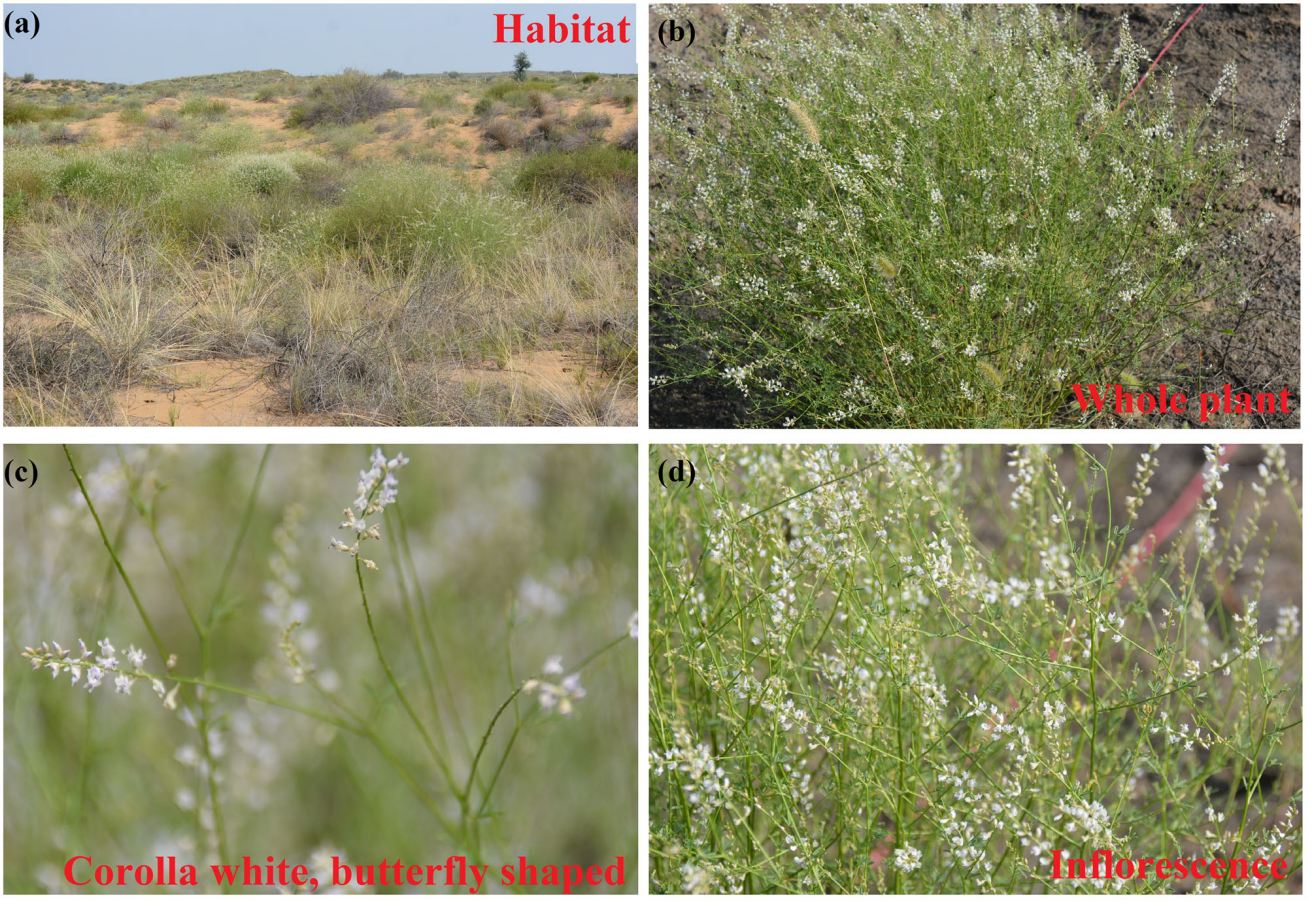
Habitat of *A. melilotoides* (a: habitat; b: the whole plant; c: corolla feature; d: infolrescence).

The sample of *A. melilotoides* was collected from semi-fixed moving dune on southwest margin of Mu Us Sandy Land, Ningxia Hui Autonomous Region, China (38°4′39.04″N, 106°32′26.27″E, alt. 1163 m), and dried with silica gel. The specimen (No. 2022ASME001LY) was deposited at the herbarium of the Institute of Forestry and Grassland Ecology, Ningxia Academy of Agriculture and Forestry Science (http://www.nxaas.com.cn/, Wangsuo Liu, email: liuwangsuo@sina.com). *A. melilotoides* is a common and excellent leguminous forage in the desert ecosystem, it is characterized by having white corolla, flag valve suborbicular, base with short stipe, wing valve slightly shorter than flag valve, apex unequal 2-lobed, base with short ear, keel valve shorter than wing valve, apex purplish, ovary subsessile, and glabrous (Fu 1993; Figure 1).

## Methods

Genomic DNAs were extracted from samples by a modified CTAB method (Stefanova et al. 2013). The genome sequencing was performed by Illumina Hiseq 2500 at Biomarker Technologies Corporation. We assembled the cp genome *via* the program SPAdes3.11.0 (Nurk et al. 2013), with that of *A. gummifer* Labill. (1790) (GenBank: MN746310) as the initial reference. The cpDNA of *A. melilotoides* was annotated using Plann (Huang and Cronk 2015). The online tool OGDRAW (https://chlorobox.mpimp-golm.mpg.de/OGDraw.html) was conducted to generate the cp genome maps (Greiner et al. 2019). To further clarify the *A. melilotoides* phylogenetic position, we downloaded the cp genome sequences of 34 *Astragalus* species in NCBI database and select two *Oxytropis* as outgroup. We aligned *A. melilotoides* and the other cp genomes by MAFFT-7.037 (Katoh and Standley 2013), and the maximum-likelihood tree was constructed by MEGA_X_10.2 with 1000 bootstrap replicates (Kumar et al. 2018). The sequence of *A. melilotoides* complete cp genome has been submitted to NCBI database (accession number ON854659).

## Results and discussion

**Figure 2.**
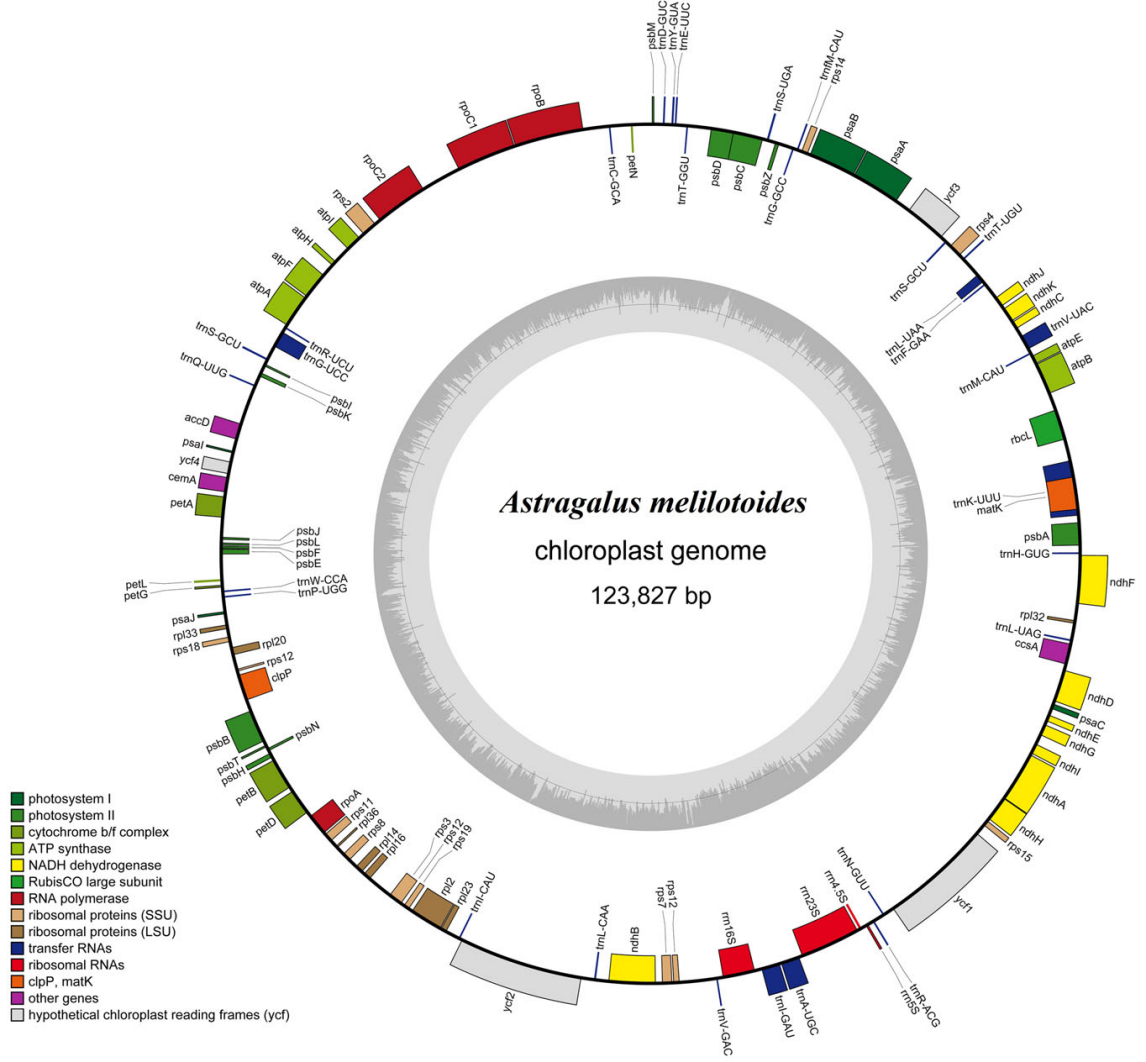
Gene map of *A. melilotoides* chloroplast genome. Genes shown inside the circle indicate that the direction of transcription is clockwise, while those shown outside are counterclockwise. Different groups of functional genes are indicated in different colors. The GC content is shown in the dashed area in the inner circle.

**Figure 3.**
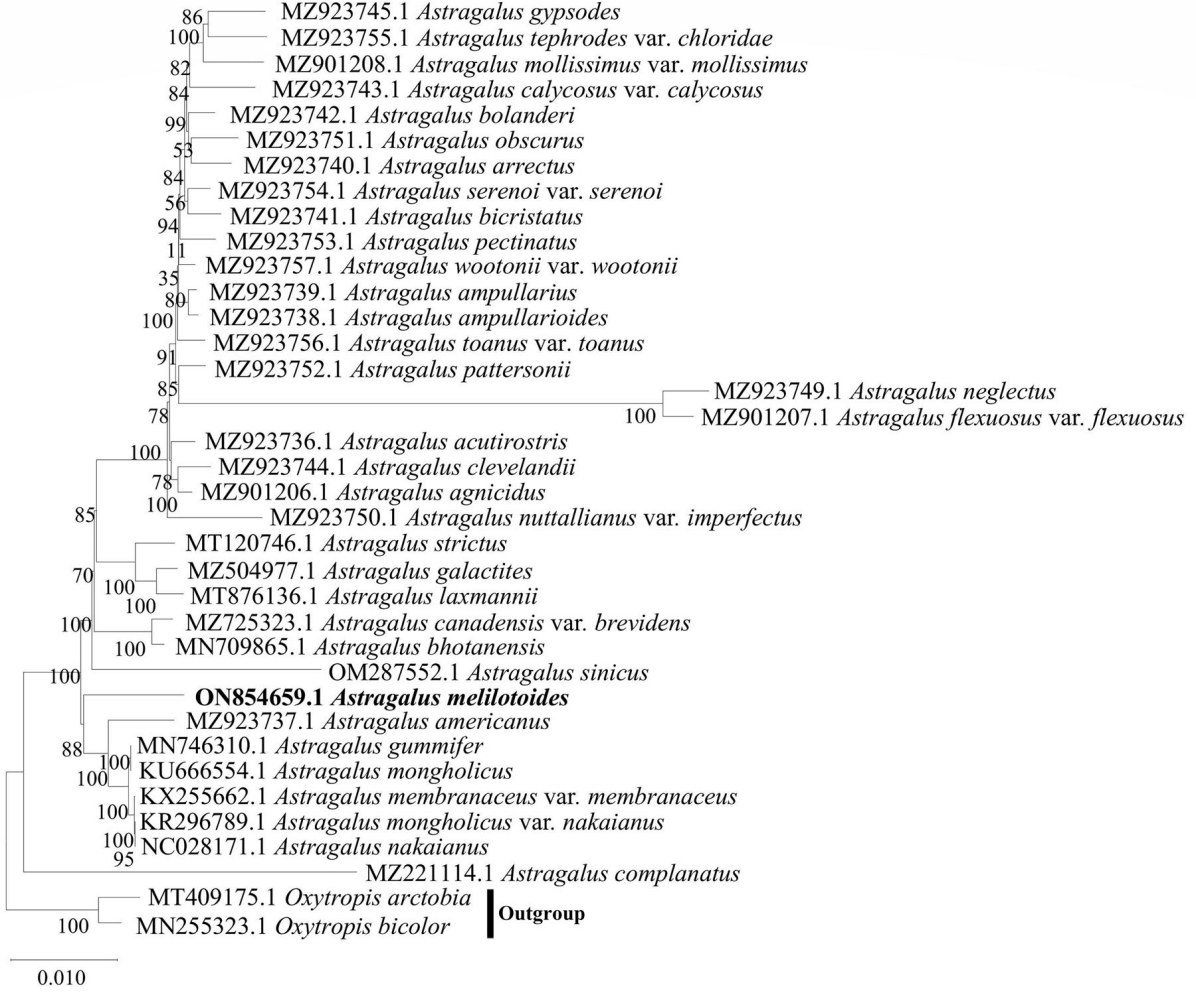
Phylogenetic relationship between *Astragalus melilotoides* and other 34 species of *Astragalus* based on the complete cp genome sequences from the NCBI database by using MEGA-X. Among this species, *A. sinicus* (OM287552) (Ke et al. 2022)*, A. galactites* (MZ504977) (Ding et al. 2021), *A. membranaceus* var*. membranaceus* (KX255662) (Wang et al. 2016)*, A. laxmannii* (MT786136) (Liu et al. 2020), and *A. complanatus* (MZ221114) (Yang 2021) have been published. *Oxytropis arctobia* and *Oxytropis* bicolor were chosen as the outgroups. The number on each node represents bootstrap values.

The complete cp genome of *A. melilotoides* was 123,827 bp in length, circular in form with IR loss, consistent with some of the species in Fabaceae (Ding et al. 2021; Guo et al. 2021; Ke et al. 2022). The cp genome encoded 110 genes, containing 76 protein-coding genes, 30 tRNA genes, and four rRNA genes. The overall GC content was 36.97% (Figure 2). The maximum-likelihood phylogenetic tree showed that *A. melilotoides* was closely related to *A. americanus*, *A. gummifer*, *A. mongholicus*, *A. nakaianus*, *A. mongholicus* var. *nakaianus*, and *A. membranaceus* var*. membranaceus* (Figure 3). Previous study for the phylogenetic characteristics of *Astragalus* were verified in the clustering tree in this study (Ding et al. 2021; Guo et al. 2021; Ke et al. 2022; Wang et al. 2016). The *A. melilotoides* complete cp genome provides valuable reference for the taxonomy and phylogenetic evolution study of *Astragalus*.

## Conclusion

This study reported that the complete cp genome of *A. melilotoides* a typical circular form with IR loss, and 123,827 bp in length. A total of 110 genes were found in the cp genome of *A. melilotoides*, including 76 protein-coding genes, 30 tRNA genes, and 4 rRNA genes, and the GC content accounted for 36.97%. The phylogenetic tree reflects *A. melilotoides* has the closely relationships with *A. americanus*, *A. gummifer*, *A. mongholicus*, *A. nakaianus*, *A. mongholicus* var. *nakaianus*, and *A. membranaceus* var*. membranaceus*. This result provides a reference for the phylogenetic study of *Astragalus* in Fabaceae family.

## Ethical approval

The collection and research process of plant material (*A. melilotoides*) was carried out in strict accordance with the guidelines provided by the herbarium of Ningxia Academy of Agriculture and Forestry Science and Chinese regulations.

## Data Availability

The data that support the findings of this study are openly available in NCBI database at https://www.ncbi.nlm.nih.gov/, reference number ON854659. The associated BioProject, SRA, and Biosample numbers are PRJNA852531, SRR19939288, and SAMN29332762, respectively.
